# Wind Exposure Regulates Water Oxygenation in Densely Vegetated Shallow Lakes

**DOI:** 10.3390/plants10071269

**Published:** 2021-06-22

**Authors:** Cristina Ribaudo, Juliette Tison-Rosebery, Mélissa Eon, Gwilherm Jan, Vincent Bertrin

**Affiliations:** 1 EA 4592 Géoressources & Environnement, F-33600 Pessac, France; 2 INRAE, UR EABX, F-33612 Cestas, France; juliette.rosebery@inrae.fr (J.T.-R.); melissa.eon@inrae.fr (M.E.); gwilherm.jan@inrae.fr (G.J.); vincent.bertrin@inrae.fr (V.B.); 3 Pôle R&D Écosystèmes Lacustres (ECLA), F-13100 Aix-en-Provence, France

**Keywords:** lake management, modelling, carbon dioxide, aquatic weeds, respiration, hypoxia, Keddy Index, submerged aquatic vegetation (SAV)

## Abstract

The presence of dense macrophyte canopies in shallow lakes locally generates thermal stratification and the buildup of labile organic matter, which in turn stimulate the biological oxygen demand. The occurrence of hypoxic conditions may, however, be buffered by strong wind episodes, which favor water mixing and reoxygenation. The present study aims at explicitly linking the wind action and water oxygenation within dense hydrophytes stands in shallow lakes. For this purpose, seasonal 24 h-cycle campaigns were carried out for dissolved gases and inorganic compounds measurements in vegetated stands of an oligo-mesotrophic shallow lake. Further, seasonal campaigns were carried out in a eutrophic shallow lake, at wind-sheltered and -exposed sites. Overall results showed that dissolved oxygen (DO) daily and seasonal patterns were greatly affected by the degree of wind exposure. The occurrence of frequent wind episodes favored the near-bottom water mixing, and likely facilitated mechanical oxygen supply from the atmosphere or from the pelagic zone, even during the maximum standing crop of plants (i.e., summer and autumn). A simple model linking wind exposure (Keddy Index) and water oxygenation allowed us to produce an output management map, which geographically identified wind-sheltered sites as the most subjected to critical periods of hypoxia.

## 1. Introduction

In lentic shallow water bodies, the diel and seasonal oxygen balance is given by the interplay between the photosynthetic activity of primary producers (net production of dissolved oxygen, DO), their respiration (net DO consumption) and heterotrophic respiration of bacteria and animals (net DO consumption). When present, submerged aquatic vegetation (SAV) induces significant diel fluctuations in oxygen levels [1]. During the day, a supersaturation of oxygen (>100%) is observed due to photosynthesis, while at night oxygen is no longer produced, and consumption processes are predominant due to respiration. This type of nycthemeral variation is particularly emphasized in summer, when plant photosynthetic rate and heterotrophic respiration are at their maximum, and whose net effect largely exceeds the contribution of temperature-dependent oxygen solubility [2,3]. Primary production releases high amounts of oxygen in the water column, allowing for the oxidation of methane (CH_4_) by methanotrophic epiphytic bacteria [4,5]. In addition, radial oxygen loss in the rhizosphere [6,7,8] contributes to reduce benthic CH_4_ flux through benthic methanotrophy or oxidation of nitrate [9]. The synthesis of large quantities of biomass occurs through the assimilation of nutrients (including N-compounds, phosphate and carbon dioxide, CO_2_); SAV is thus able to uptake gases and nutrients coming from the sediment and the atmosphere, and synthesize them in biomass [10]. However, oxygen dynamics can be altered in densely vegetated stands, such as those dominated by invasive macrophytes: here, due to a fast cycle of growth and decay, fresh organic matter continuously replenishes the organic bulk in the sediment [11]. In those conditions, elevated biological oxygen demand (BOD) and hypoxia are coupled to accumulation and stratification of nutrients, such as carbon dioxide, methane, ammonium and reactive phosphorous, also during the day [12,13,14,15].

Dense plant canopies are known to locally generate bottom shading and modify water circulation, that impeding convective cooling, even within small depths [16,17,18,19,20]. This can affect or, inversely, exacerbate the thermally driven exchange flow of nutrients and DO between pelagic and littoral zones of the lake [21]. Nevertheless, it has been assessed that wind episodes may induce surface and internal flows even within stratified waters [22]. Shallow lakes are continuously subjected to mixing and wave-breaking in function of wind speed [21]; in function of the lake size, slope and bathymetry, wind-exposed lakes are also concerned by periodic seiche events which contribute to water mixing [23,24]. Still, only few studies infer that wind action may induce local turbulent mixing and reaeration even within dense submerged canopies [14,18]. Indeed, the explicit interaction between wind exposure and ecosystem functioning in shallow plant-dominated lakes remains almost undescribed [16,25], despite the growing demand for precision by stakeholders (users, managers, politicians) in the domain of biological invasions.

Invasive aquatic plants management is one of the main issue concerning global changes in freshwaters [26]. In the past, manager’s decisions on invaded environments were mainly driven by socio-economical questions relating to tourism, boating and swimming [27]. Recently, invasive plants management started to progressively embrace ecological sciences, with the primary goal of understanding if invaded sites are *de facto* degraded or imperiled regarding to their functioning. As a result, aquatic weeds are more and more in question about their overall role on ecosystem metabolism. Coherently, managers are demanding to ecologists to produce effective and readable tools for ameliorating their interventions [28,29]. We herein report a study specifically addressing this issue, which employs DO saturation as a reliable indicator of net ecosystem metabolism, related to autotrophic and heterotrophic processes [30]. Firstly, we hypothesize that the impact on DO levels within invasive macrophyte stands significantly differs in function of the degree of wind exposure. Secondly, we hypothesize that DO levels are inversely correlated to plant densities and sedimentary organic matter content. Thirdly, we propose a quantitative tool to spatially identify sites that are more risky for hypoxic events, and thus need intervention by managers.

## 2. Results

### 2.1. Diel Variations in Vegetated Stands

**Figure 1:**
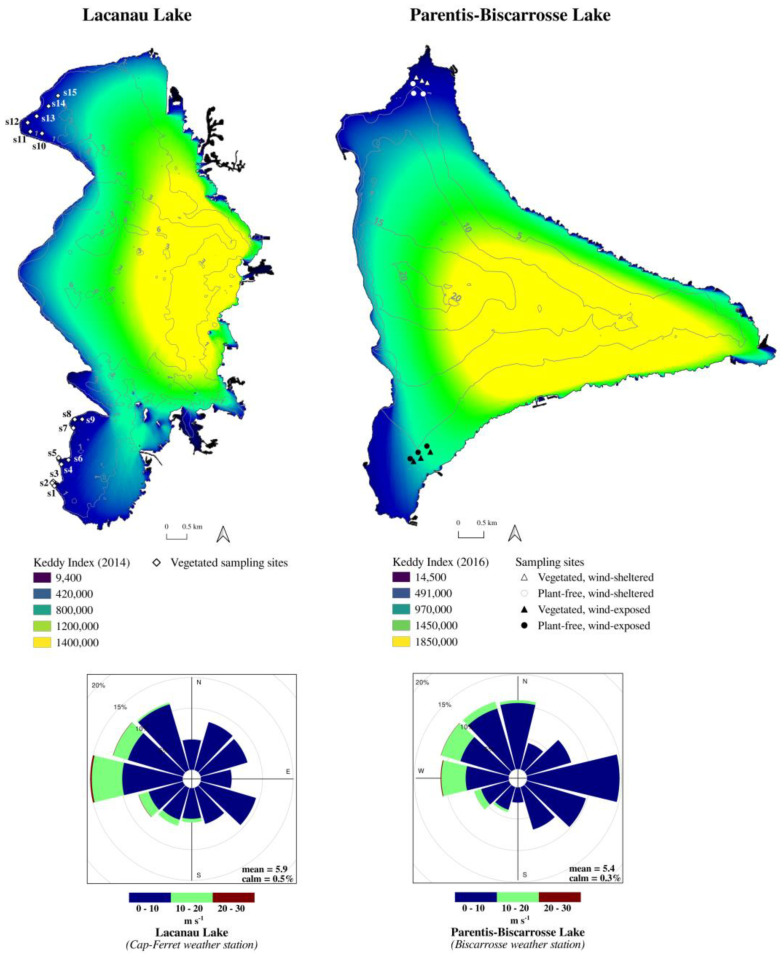
Keddy Index calculated on annual basis for Lacanau Lake (on the **left**) and Parentis-Biscarrosse Lake (on the **right**). The windrose is calculated on wind speed and direction hourly data on an annual basis. Lake bathymetry and sampling sites for seasonal 24-cycle campaigns (LAC Lake), as well as sampling sites for seasonal campaigns at wind-sheltered and -exposed sites (PAR Lake) are reported.

Sampling sites in Lacanau Lake (hereafter, LAC Lake) were homogeneously distributed within the largest invasive macrophyte stands of the lake, which developed in the most sheltered zones of the lake (Figure 1).

**Figure 2:**
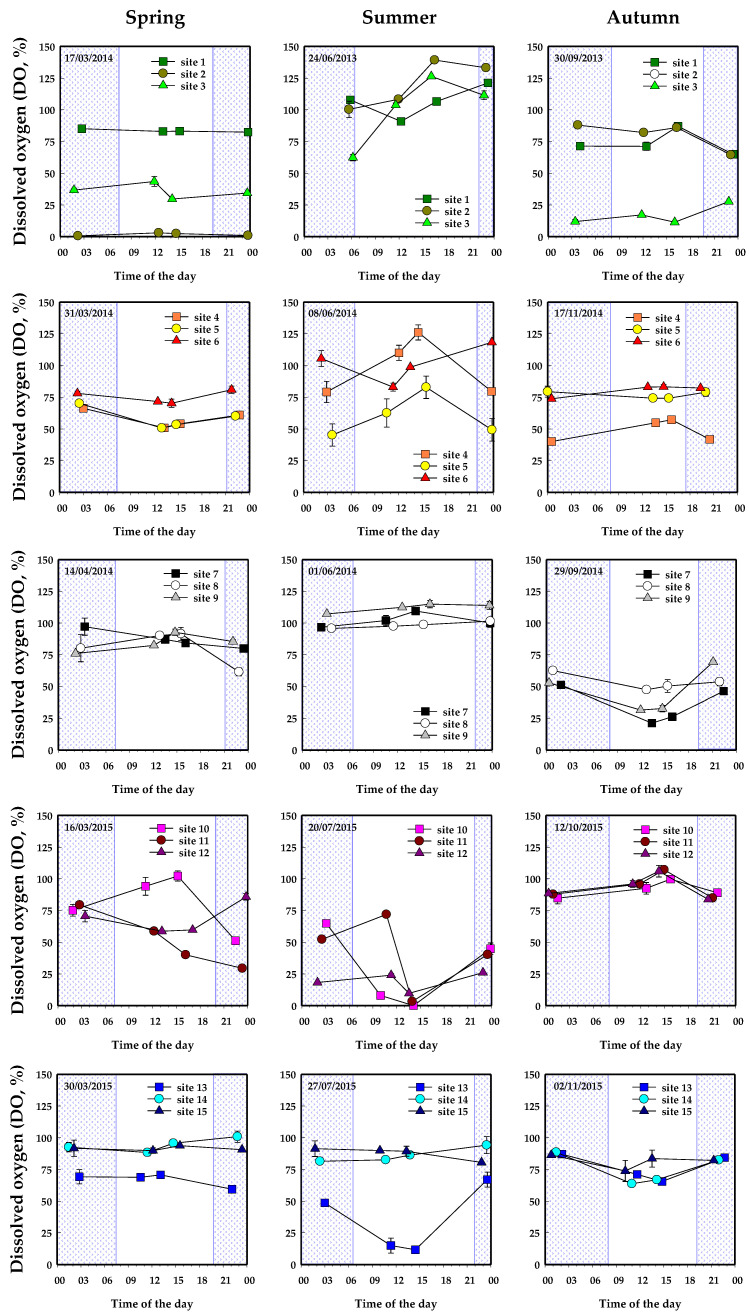
DO results from seasonal 24 h-cycle campaigns in LAC Lake. Measurements were carried out within densely vegetated areas, presenting biomass >100 g_DW_ m^−2^ during samplings. Hatched color indicates night periods.

Results from seasonal 24 h-cycle campaigns showed that most of the sites were hypoxic (DO saturation <100%), and that DO depletion also occurred during daylight (Figure 2). Concomitantly, CO_2_ was mostly supersaturated and pH acid (pH < 7), with some exceptions during daylight in summer and autumn (Figures S1 and S2). CH_4_, NH_4_^+^ and NO_3_^−^ buildup in the water column appeared both during the night and day (Figures S3–S5). Water temperature ranged from 11.1 ± 0.2 to 26.7 ± 0.3 °C (in spring and summer, respectively—Figure S6) and DOC averaged 13.1 ± 0.2 mg L^−1^ on an annual basis.

ANOVA test revealed that, with temperature and ammonium as solely exceptions (day > night), dissolved gases and inorganic compounds measured within plant stands did not differ between day and night; all parameters varied seasonally (Table 1).

### 2.2. Wind-Sheltered vs. Wind-Exposed Sites

The choice of sampling sites in Parentis-Biscarrosse Lake (hereafter, PAR Lake) was based on two co-occurring conditions: the presence of densely vegetated areas and the difference in wind exposure (Figure 1). Results from seasonal campaigns in PAR Lake showed that dissolved gases and inorganic compounds significantly changed in function of wind exposure (ANOVA, Table 2).

**Figure 3:**
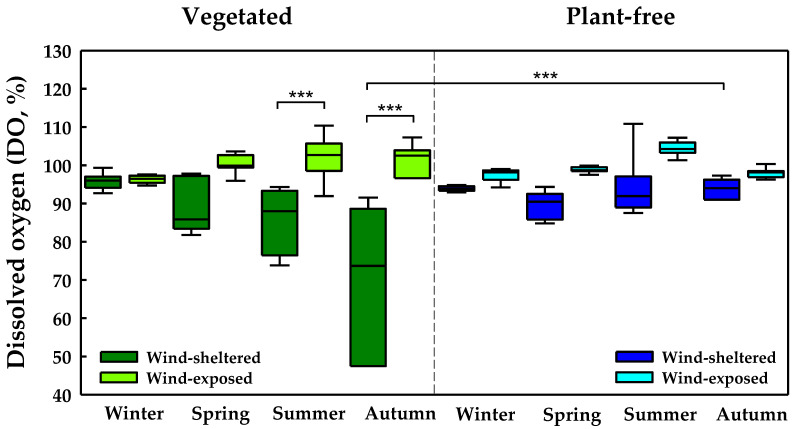
DO results from seasonal campaigns in PAR Lake. Measurements were carried out at wind-sheltered and -exposed sites, in vegetated and plant-free areas. For a better readability, Tukey’s HSD results are not reported for the seasonality factor. *** indicates *p*-value < 0.001.

Differences between sheltered and exposed sites were significant for every physicochemical parameter, yet only at vegetated sites and in function of the season (in summer and in autumn). Significant differences between vegetated and plant-free sites occurred only at sheltered sites. Tukey’s HSD test indicated that water temperature was lower at sheltered sites than at exposed ones (Figure S7). pH values were lower at vegetated and sheltered sites than at exposed ones, only during summer (Figure S8). DO was lower at vegetated and sheltered sites than at exposed ones (Figure 3); CO_2_ and CH_4_ were higher at vegetated and sheltered sites than at exposed ones (Figures S9 and S10); NH_4_^+^ and NO_3_^−^ values differed seasonally between sheltered and exposed sites, with no differences between vegetated and plant-free areas (Figures S11 and S12). DOC averaged 6.2 ± 0.2 mg L^−1^ on an annual basis.

### 2.3. Dependence of DO Saturation on Plant Biomass and Sedimentary OM

Total biomass varied seasonally at both lakes, with values comprised between 319 ± 245 and 668 ± 414 g_DW_ m^−2^ at LAC Lake (in spring and summer, respectively), and between 1626 ± 132 and 4528 ± 2413 g_DW_ m^−2^ at PAR Lake (in spring at exposed and in autumn at sheltered sites, respectively). OM content in vegetated sediments ranged from 0.7 ± 0.2 to 71 ± 3% and from 0.7 ± 0.1 to 1.2 ± 0.1% as LOI, for LAC Lake and PAR Lake, respectively. Linear mixed-effects model, calculated on the two lakes dataset, showed that DO saturation was not dependent on OM sedimentary content on the total plant biomass; only DO values measured in LAC Lake during summer resulted in being negatively correlated to biomass (*p*-value < 0.01).

### 2.4. Dependence of DO on Wind Exposure and Hypoxia Risk Map Production

**Figure 4:**
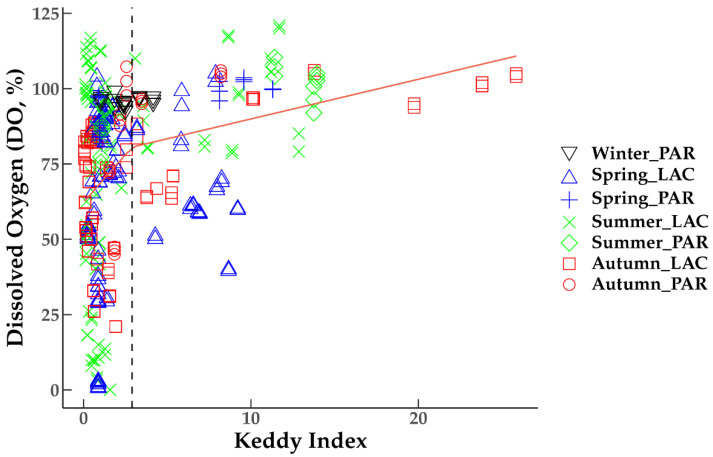
Dependence of DO saturation on wind exposure (Keddy Index). Dashed line is the breakpoint indicating the threshold between low-risk and high-risk of hypoxia/anoxia events in densely vegetated areas.

The regression of DO saturation against wind exposure, identified with the segmented function in R, showed a structural breakpoint at Keddy Index = 2.9 (Figure 4). We considered this breakpoint as a threshold of hypoxia risk, i.e., low risk above this value and high risk below. This threshold is assumed to be the minimum wind exposure which would be able to decrease the risk of hypoxia in dense submerged plant stands.

**Figure 5:**
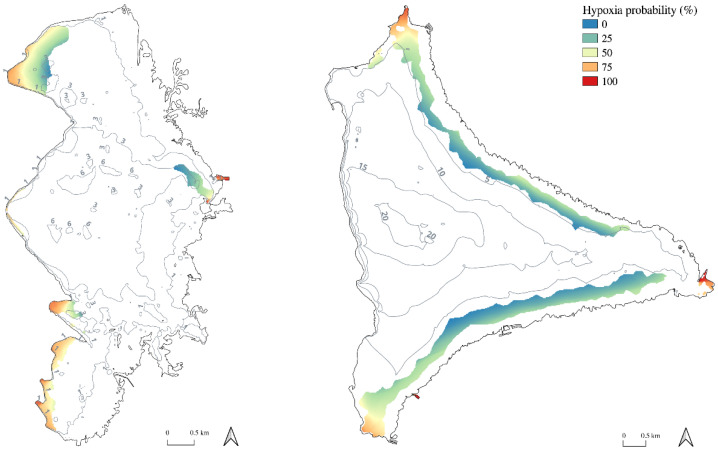
Hypoxia risk maps calculated for one year in densely vegetated areas of LAC Lake (on the **left**) and PAR Lake (on the **right**). Lakes bathymetry is also reported.

Further, in order to produce a hypoxia risk map, Keddy Index was calculated for each 4 h-long period (*n* = 2190) on each pixel cell (*n* = 4031 for LAC Lake and *n* = 14,438 for PAR Lake) matching with densely vegetated areas, presenting biomass >50 g_DW_ m^−2^, mapped at the lake scale (1.19 km^2^ and 4.17 km^2^ in LAC and PAR Lakes, respectively, from 31) (Figure 5). Hypoxia risk was above 50% in 70 ha of plants stands (corresponding to 60% of the total vegetated surface) in LAC Lake and in 50 ha in PAR Lake (12% of the total vegetated surface). This risk was above 75% in 11 ha of plants stands (9% of the total vegetated surface) in LAC Lake and in 11ha in PAR Lake (3% of the total vegetated surface).

## 3. Discussion

In vegetated stands, diel variations of inorganic compounds typically reflect plants photosynthetic activity, with the lowest dissolved carbon and nitrogen concentrations measured in the water at late afternoon, corresponding to nutrients depletion by plants uptake, then an accumulation during the night, with a peak just before dawn. At the same time, DO and pH follow an exactly inverse pattern. In our study, the described nycthemeral shape was detectable only at some sites and mostly during summer. At other sites, heterotrophic activity, stimulated by temperature increase during summer and autumn, exceeded net oxygen release during the day, that resulting in hypoxia/anoxia events and buildup of CO_2_, CH_4_ and NH_4_^+^ in the water column. This observation is recurrent in dense stands formed by invasive macrophytes, where the sedimentation of organic matter generates an elevated benthic BOD during the period of senescence of plants; this implies a permanent DO deficit [15,31,32]. In dense hydrophyte stands, DO input from the atmosphere can be limited to the surficial layer of the water column, as long stems constitute a physical barrier, as floating-leaved macrophytes do [13]. In our case, the occurrence of a vertical “plant wall” at the external boundaries of vegetated stands may also lead to the annihilation of the horizontal flow of nutrients and DO from the pelagic to the littoral zones [22].

Hypoxic events and inorganic compounds buildup can be however contrasted by the wind action, which may induce local turbulent mixing and reaeration even within dense submerged canopies [14,18]. Coherently, some of the diel variations measured in our study showed a flattened shape, with constant values along the 24 h-cycle. On one hand, elevated DO values during the night could be attributable to convective mixing due to air temperature nightly decrease [17,33]. On the other hand, the maintaining of constant DO values along a diel cycle may be an indicator of stationary wind conditions and turbulent mixing; this supposition is supported by the second part of our study. Seasonal campaigns at wind-sheltered and -exposed sites showed that, ecosystem functioning was not ascribable to the plant presence/absence or to the seasonal biomass variation only. Indeed, DO and CO_2_ saturation at wind-exposed sites hovered at about 100% all year round, indicating that wind-driven diffusion continuously outreached net production and consumption within the water column, even in invaded areas of the lake. Overall results show thus that the presence of invasive hydrophytes does not systematically promote water hypoxia, if local wind conditions allow an efficient water mixing by wind.

When considering the whole dataset, only DO values measured in LAC Lake during summer resulted in being dependent on plant density; moreover, vegetated stands in this lake mainly developed at sheltered sites [34]. Prevailing winds oriented from the northwest created low hydrodynamic conditions, because of the natural barrage formed by sand dunes [35]. Elevated plant biomass matching with shallow depths in wind-sheltered areas seemed to generate favorable conditions for water hypoxia, a phenomenon exacerbated by an elevated turnover of biomass during summer. In contrast, extremely elevated biomass measured in PAR Lake, largely exceeding values reported until now for *Egeria* spp. invaded sites [3,31], did not generate an extreme DO deficit even at wind-sheltered sites. The difference between the two lakes is evident also from a thermic point of view: at LAC Lake, a previous study had showed that water temperature measured in vegetated stands was significantly lower than that measured in plant-free areas, irrespective of the season [19]. The present study on PAR Lake shows instead that no significant difference exists between vegetated and plant-free areas, irrespective of the season (Figure S7). As for the DO and CO_2_, the divergence in temperature results among the two lakes could be due to the different size of the lake, the second being larger and permitting fetch length—and thus, water mixing—to be more important.

The hypoxia risk map shows that elevated hypoxia probability is associated with wind-sheltered areas of the lakes, and that oxygenation shortage can affect a large total surface of several tens of hectares. Hypoxia risk is at its maximum in both lakes at enclosed and wind-sheltered areas, like small marinas and public boat launches, which are known to be important drivers of aquatic plant spread [36,37]. On the other hand, large surfaces of the lake invaded by elevated plant densities would not be affected by hypoxia and would thus not necessitate intervention. The hypoxia risk map we produced represents a preliminary and concrete tool, coupling field measurements and modelling, which can reduce plant management costs, as it indicates precisely where invasive plants constitute a problem for ecosystem functioning. A similar approach providing reproducible management tools has been recently published [38], that coupling lake depth or bathymetry to anoxia probability in the hypolimnion of deep lakes. Our model should be, however, calibrated site-specifically, because the intrinsic sedimentary features and the trophic status of the lake could affect the magnitude of hypoxia level and nutrients flux. The two lakes we studied presented different DOC values, sedimentary OM content and resulted in very different concentrations of CO_2_ and CH_4_. Also, due to a different fetch length, the reaeration strongly varied even within comparable wind velocity. A possible improvement of our method could have been to introduce the local bathymetry in the model. Indeed, waves induce vertical upward forces acting on the water column movements and sediment resuspension [34]; furthermore, wind-induced circulation in nearshore zones appears to be crucial in littoral plant-free areas [24]. We can expect an increase of wind effect on water mixing in shallow zones due to orbital movements translated to the lake bottom. Nevertheless, SAV also reduce waves action and current velocities within beds [39,40]. Future modelling works should thus focus on integrating vegetation in the photic region to better define how the cross-shore water circulation works. Another possible improvement in the future would be the use of automatic oxygen probes, in order to obtain a finer resolution scale of diel and seasonal variations, and perfect the calculation of hypoxia risk probability on a long temporal scale.

Our results highlight the need to consider local hydrodynamics in lake management decisions. Wind exposure should be used for spatially organizing management plans and prioritizing zones where invasive biomass control actions are needed. Mapping hypoxia risk in densely vegetated stands is a promising tool for the management of invasive hydrophytes in shallow lakes.

## 4. Materials and Methods

### 4.1. Study Area

Lacanau Lake and Parentis-Biscarrosse Lake are shallow lakes located in the southern Atlantic coast of France. Those lakes are characterized by sandy acidic substrate and classed as oligo-mesotrophic (Lacanau, 16 km^2^) and eutrophic (Parentis-Biscarrosse, 32 km^2^). Within the two lakes, large submerged stands of *Egeria densa* Planch. and *Lagarosiphon major* (Ridl.) Moss develop between 1 and 7 m deep, with dense stands being preferentially located at shallow and wind-sheltered sites, or at deep and wind-exposed sites [34].

### 4.2. Field Campaigns

Between June 2013 and November 2015 at Lacanau Lake, seasonal 24 h-cycle campaigns were carried out at 15 sites. Sampling sites were homogeneously distributed within the largest invasive macrophyte stands of the lake [34]. Water was collected within plant canopy at depths ranging from 100 to 330 cm, with a frequency of four times a day (two samplings during the day, between 11 a.m. and 3 p.m.; two samplings during the night, between 9 p.m. and 6 a.m.). Water temperature (T, °C), pH, dissolved oxygen (DO, expressed as saturation %), dissolved carbon dioxide (CO_2_, %), dissolved methane (CH_4_, µM), nitrate (NO_3_^−^, µM), ammonium (NH_4_^+^, µM) and dissolved organic carbon (DOC, mg L^−1^) were measured according methods reported in [19]. Finally, we tested the influence of the sampling time on the biogeochemistry of the water column by a two-way ANOVA with interactions among factors. The diel variation (two levels: day vs. night) and the season (three levels: spring vs. summer vs. autumn) were considered as fixed factors, while the sampling site (fifteen levels) was considered as a random factor.

Between January 2016 and January 2017 at Parentis-Biscarrosse Lake, seasonal sampling campaigns were carried out, during the day only, at vegetated (3 wind-sheltered and 3 wind-exposed sites) and at plant-free sites (3 wind-sheltered and 3 wind-exposed). The degree of wind exposure was estimated by previous modeling of wind exposure Keddy Index [41]. Water was collected within plant canopy at depths ranging from 150 to 300 cm, between 11 a.m. and 3 p.m. Water samples collection, treatment and analyses are the same as those adopted in Lacanau Lake and reported in [19]. Finally, we tested the influence of spatial exposure to wind on the biogeochemistry of the water column by a three-way ANOVA with interactions among factors. The degree of wind exposure (two levels: exposed vs. sheltered), the plant presence (two levels: vegetated vs. plant-free) and the season (four levels: winter vs. spring vs. summer vs. autumn) were considered as fixed factors, while the sampling site (twelve levels) was considered as a random factor.

Normal distribution (Shapiro–Wilk Test) and homoscedasticity (Levene’s Test) were tested before running ANOVAs. Post hoc analyses were performed by Tukey’s Honestly Significant Difference (HSD) test. Statistical analyses were performed with R Program [42]. Mean values are reported with their standard deviation.

Macrophytes sampling was carried out by rake for total biomass (g_DW_ m^−2^) measurements, immediately after water samplings, as reported in [19]. Concomitantly, within the plant stands, sediment samples were collected by grabber, as described in [34], for sedimentary organic matter (OM, as loss of ignition, % LOI) measurements. In order to test the dependence of DO saturation on plant biomass and OM content, a linear mixed-effects model fit by maximum likelihood was performed on the whole dataset (DO measurements from Lacanau and Parentis-Biscarrosse Lakes), with the sampling site as a random factor.

### 4.3. Wind Exposure Calculations

Wind exposure was calculated according [41] for both lakes by using a fetch matrix (i.e., the distance over which waves can build up) obtained from lake open-water raster grid cells (resolution of 17 m) for each wind compass direction (10–360°, in 10° increments). Wind data (hourly and daily mean speed and direction) were provided by Météo France in Cap-Ferret (44°37′54″ N, 1°14′53″ O) and Biscarrosse (44°25′54″ N, 1°14′51″ O) weather stations for Lacanau and Parentis-Biscarrosse Lakes, respectively. It is possible to generate values which should be related to the effect of wind at a given point (here, a grid cell) by using fetch and wind velocity. For a given compass direction, one measure of exposure is the product of mean wind speed and direction and the percent frequency of the wind blowing in that direction.

In order to position wind-sheltered and -exposed sampling sites in Parentis-Biscarrosse Lake, daily mean wind speed and direction were used to build a wind exposure map during 1-year period (2014). One measure of exposure was calculated for each grid cell over 36 compass wind directions according to a fetch matrix. A cell’s total exposure is given by the sum of values calculated for all of the compass directions during the 1-year period. Sampling sites were chosen within lake areas identified as low- or highly-exposed to wind action.

### 4.4. Coupling DO and Wind Exposure

Keddy Index was calculated for the 4 h before the exact timing of water sampling. Then, each DO value was coupled to the sum of Keddy Index values for this period. This is the duration estimated being necessary for the water mixing at low depth [17,43]. In order to test the dependence of DO saturation on wind exposure, a Chow test was performed to determine the presence of a structural break at some point of the data series [44]. We used the *sctest* function from the *strucchange* package in R software to perform a Chow test, which resulted in *F* = 10.7, *p*-value = 2.7 × 10^−5^. The significance of the test indicates that a structural breakpoint is present in the regression. Or else, that two regression lines can fit the pattern in the data more effectively than a single regression line. Finally, we applied the *segmented* function in R to analyze segmented relationships in the regression, in order to obtain a breakpoint value.

### 4.5. Hypoxia Risk Map Production

We calculated 4 h-long Keddy Index values each day during one year (2014 and 2016 for Lacanau and Parentis-Biscarrosse Lakes, respectively) for each raster cell corresponding to densely vegetated areas of the lake, and presenting a biomass >50 g_DW_ m^−2^ [34]. Each 4 h-long period and each grid cell in which wind exposure was under the breakpoint value, indicating a high risk of hypoxia, was classified as “1”, whereas 4 h-long period with low risk of hypoxia (>2.9) were classified as “0”. The probability of hypoxia was expressed as the percentage (0–100%) of 4 h-long periods where wind exposure was below the hypoxia threshold during one year. Finally, this probability was reported on raster grid cells to map the hypoxia risk at densely vegetated areas scale.

## Figures and Tables

**Table 1:** Summarized results of the two-way ANOVA on physicochemical parameters (diel variation and season as fixed factors; sampling site as random factor). Results refer to seasonal 24 h-cycle campaigns carried out at 15 vegetated sites of LAC Lake.

	T (°C)		pH Units		DO (%)			
Source	df, Residuals	*p*-Value	df, Residuals	*p*-Value	df, Residuals	*p*-Value		
Diel variation	1, 204	<0.001	1, 204	n.s.	1, 383	n.s.		
Season	2, 204	<0.001	2, 204	<0.001	2, 383	<0.001		
Diel × Season	2, 204	n.s.	2, 204	n.s.	2, 383	n.s.		
	**CO_2_ (%)**		**CH_4_ (µM)**		**NH_4_^+^ (µM)**		**NO_3_^−^ (µM)**	
	**df, Residuals**	***p*** **-Value**	**df, Residuals**	***p*** **-Value**	**df, Residuals**	***p*** **-Value**	**df, Residuals**	***p*** **-Value**
Diel variation	1, 347	n.s.	1, 415	n.s.	1, 410	<0.05	1, 394	n.s.
Season	2, 347	<0.001	2, 415	<0.001	2, 410	<0.05	2, 394	<0.001
Diel × Season	2, 347	n.s.	2, 415	n.s.	2, 410	n.s.	2, 394	n.s.

**Table 2:** Summarized results of the three-way ANOVA on physicochemical parameters (wind exposure, plant presence and season as fixed factors; sampling site as random factor). Results refer to seasonal campaigns carried out at 12 vegetated and plant-free sites of PAR Lake. Please refer to figures for Tukey’HSD test differences between treatments.

	T (°C)		pH Units		DO (%)			
Source	df, Residuals	*p*-Value	df, Residuals	*p*-Value	df, Residuals	*p*-Value		
Wind exposure	1, 8	<0.001	1, 8	<0.001	1, 8	<0.001		
Plant presence	1, 8	n.s.	1, 8	n.s.	1, 8	n.s.		
Season	3, 24	<0.001	3, 24	<0.001	3, 72	<0.05		
Wind × Plant	1, 8	n.s.	1, 8	<0.05	1, 8	<0.05		
Wind × Seas	3, 24	<0.001	3, 24	<0.001	3, 72	<0.001		
Plant × Seas	3, 24	n.s.	3, 24	n.s.	3, 72	<0.05		
Wind × Plant × Seas	3, 24	n.s.	3, 24	n.s.	3, 72	<0.001		
	**CO_2_ (%)**		**CH_4_ (µM)**		**NH_4_^+^ (µM)**		**NO_3_^−^ (µM)**	
	**df, Residuals**	***p*** **-Value**	**df, Residuals**	***p*** **-Value**	**df, Residuals**	***p*** **-Value**	**df, Residuals**	***p*** **-Value**
Wind exposure	1, 8	<0.001	1, 8	<0.001	1, 8	n.s.	1, 8	<0.05
Plant presence	1, 8	n.s.	1, 8	n.s.	1, 8	n.s.	1, 8	n.s.
Season	3, 72	<0.001	3, 72	<0.001	3, 72	<0.001	3, 72	<0.001
Wind × Plant	1, 8	<0.05	1, 8	n.s.	1, 8	n.s.	1, 8	<0.05
Wind × Seas	3, 72	<0.001	3, 72	<0.001	3, 72	<0.001	3, 72	<0.001
Plant × Seas	3, 72	<0.001	3, 72	<0.05	3, 72	n.s.	3, 72	n.s.
Wind × Plant × Seas	3, 72	<0.001	3, 72	<0.05	3, 72	n.s.	3, 72	n.s.

## Data Availability

The data presented in this study are available in the article or supplementary material.

## Supplementary Materials

The following are available online at https://www.mdpi.com/article/10.3390/plants10071269/s1, Figure S1: CO_2_ results from seasonal 24 h-cycle campaigns in Lacanau Lake; Figure S2: pH results from seasonal 24 h-cycle campaigns in Lacanau Lake; Figure S3: CH_4_ results from seasonal 24 h-cycle campaigns in Lacanau Lake; Figure S4: NH_4_^+^ results from seasonal 24 h-cycle campaigns in Lacanau Lake; Figure S5: NO_3_^−^ results from seasonal 24 h-cycle campaigns in Lacanau Lake; Figure S6: Water temperature results from seasonal 24 h-cycle campaigns in Lacanau Lake; Figure S7: Water temperature results from seasonal campaigns in Parentis-Biscarrosse Lake; Figure S8: pH results from seasonal campaigns in Parentis-Biscarrosse Lake; Figure S9: CO_2_ results from seasonal campaigns in Parentis-Biscarrosse Lake; Figure S10: CH_4_ results from seasonal campaigns in Parentis-Biscarrosse Lake; Figure S11: NH_4_^+^ results from seasonal campaigns in Parentis-Biscarrosse Lake; Figure S12: NO_3_^−^ results from seasonal campaigns in Parentis-Biscarrosse Lake. In S7, S8, S9, S10, S11 and S12, Tukey’s HSD results are not reported for the seasonality factor. *** indicates *p*-value < 0.001, ** *p*-value < 0.01, * *p*-value < 0.05.
