# Supplementary figures and images for: Wind Exposure Regulates Water Oxygenation in Densely Vegetated Shallow Lakes
Source: Plants (Basel). 2021 Jun 22;10(7):1269. doi: 10.3390/plants10071269 (PMC8309138; doi:10.3390/plants10071269)

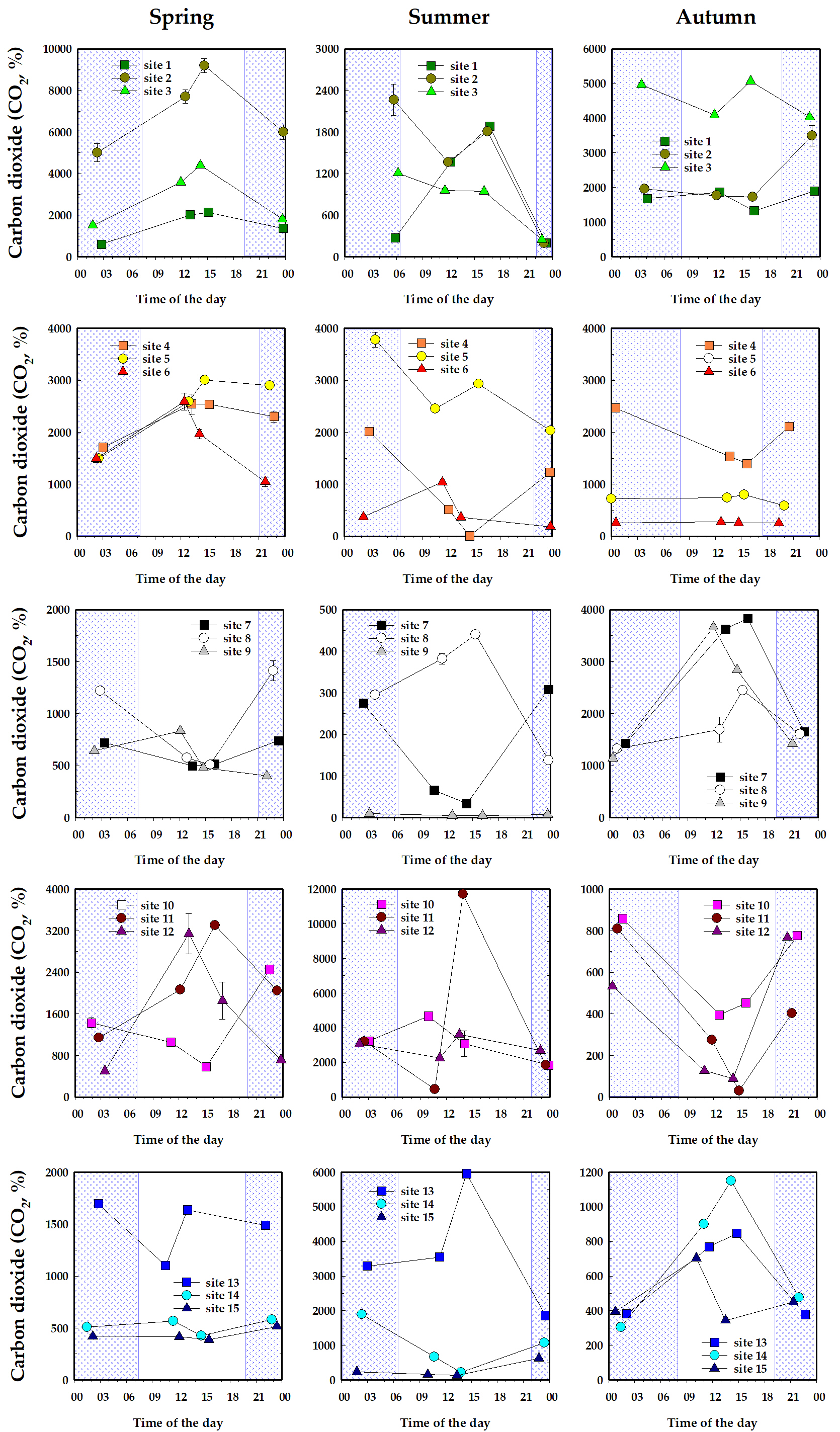

Supplement: Supplementary file 1 [file plants-10-01269-s001.zip › Figure S1.JPG]

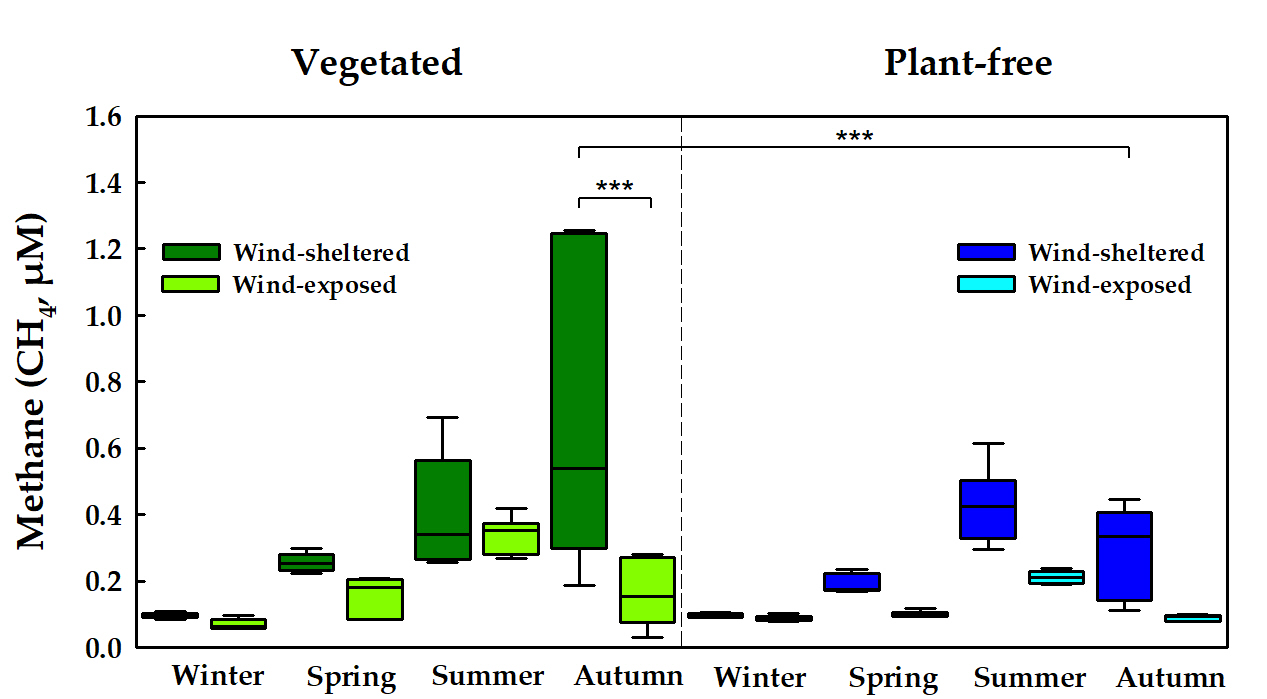

Supplement: Supplementary file 1 [file plants-10-01269-s001.zip › Figure S10.JPG]

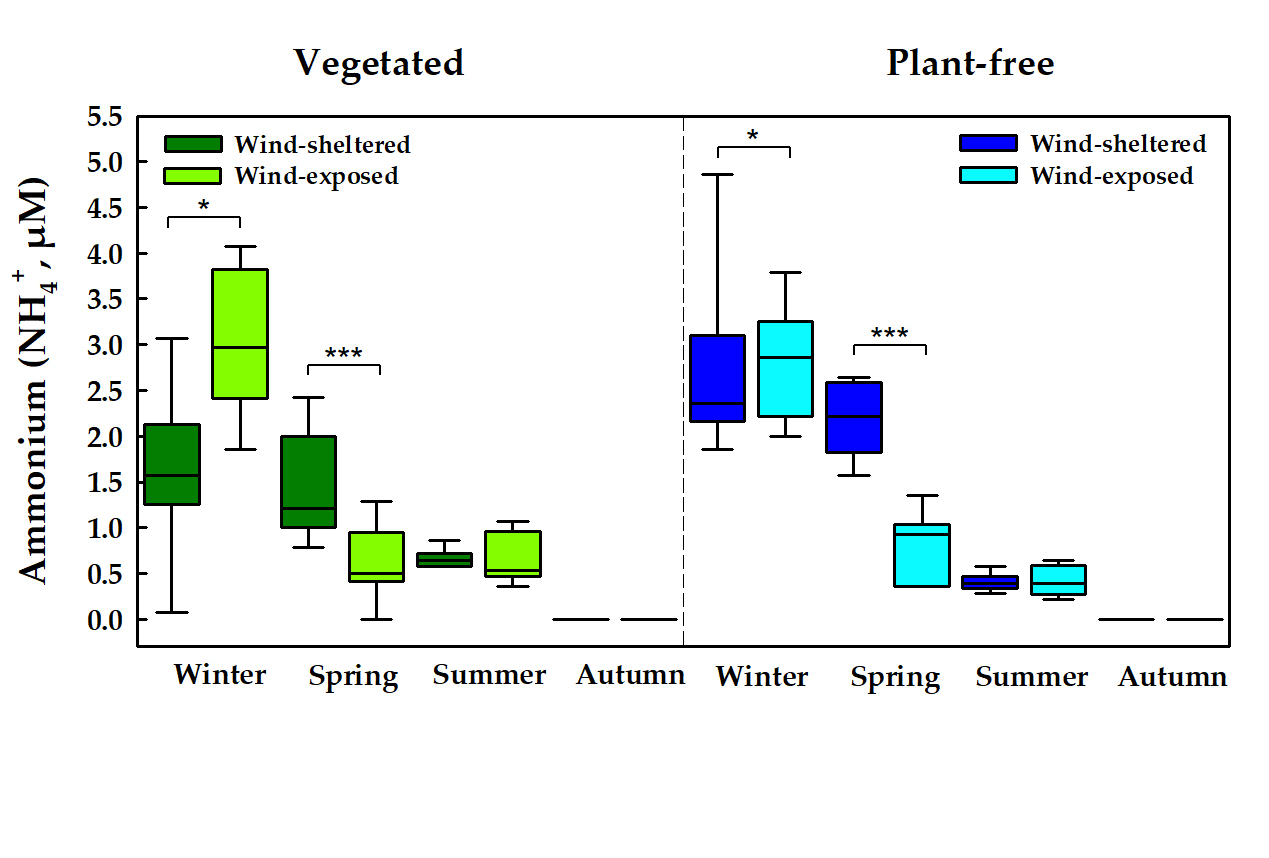

Supplement: Supplementary file 1 [file plants-10-01269-s001.zip › Figure S11.JPG]

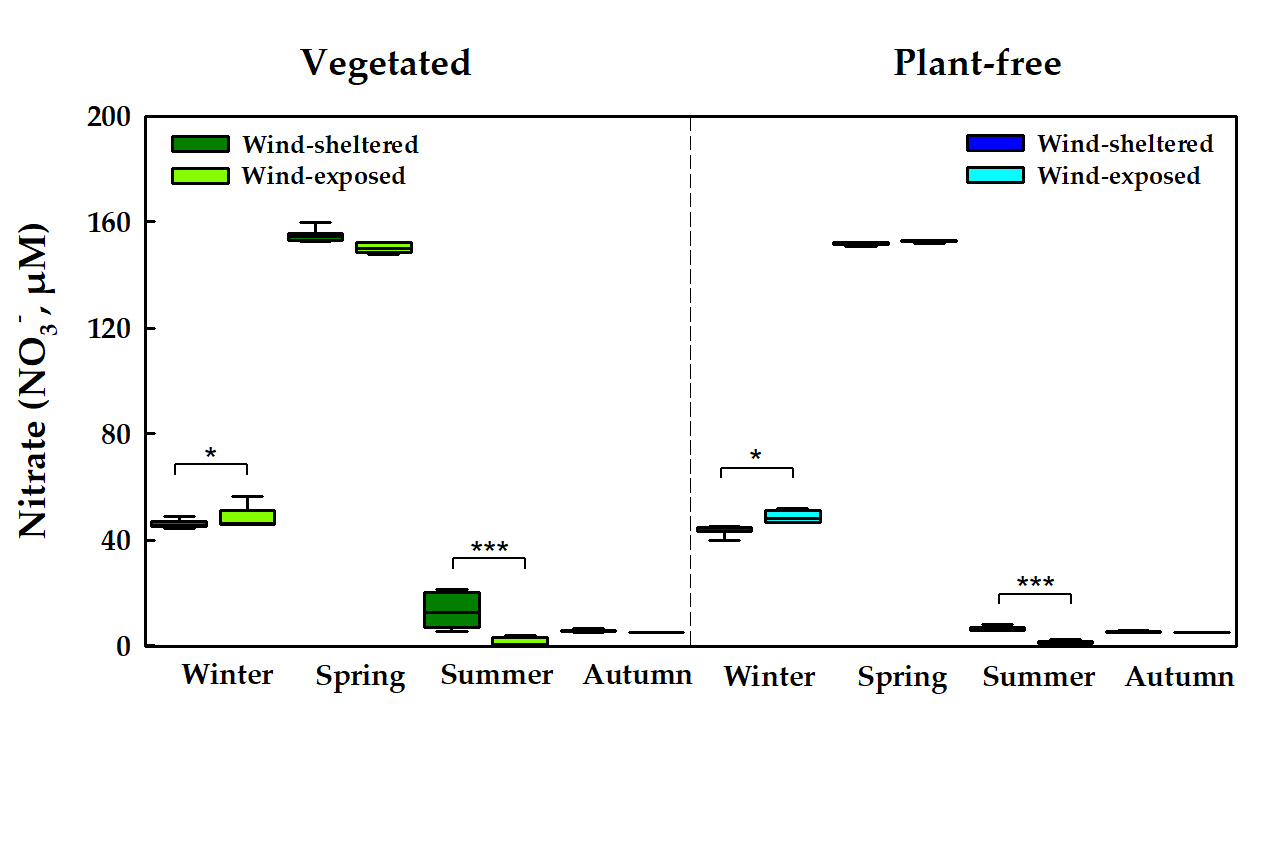

Supplement: Supplementary file 1 [file plants-10-01269-s001.zip › Figure S12.JPG]

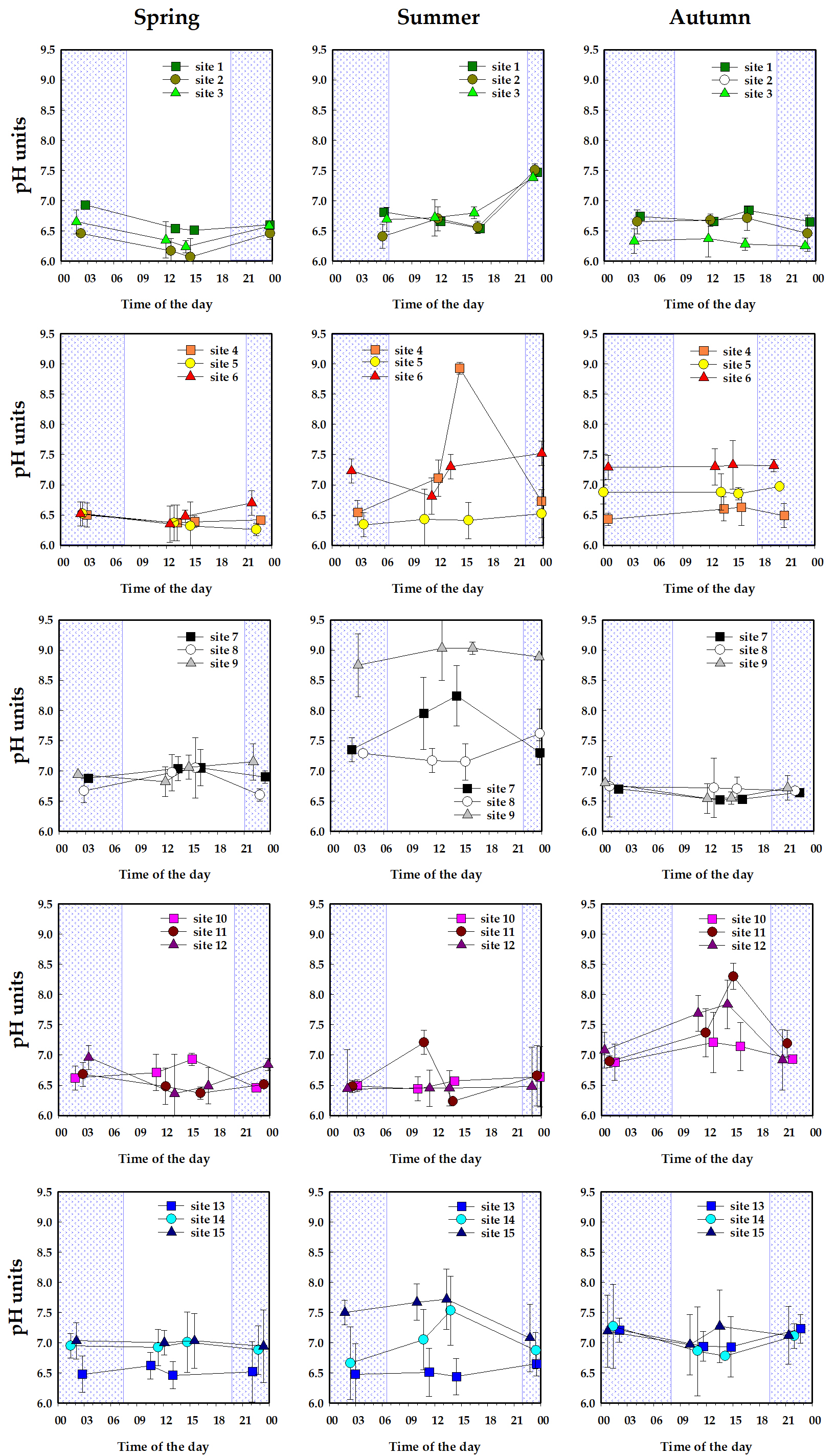

Supplement: Supplementary file 1 [file plants-10-01269-s001.zip › Figure S2.JPG]

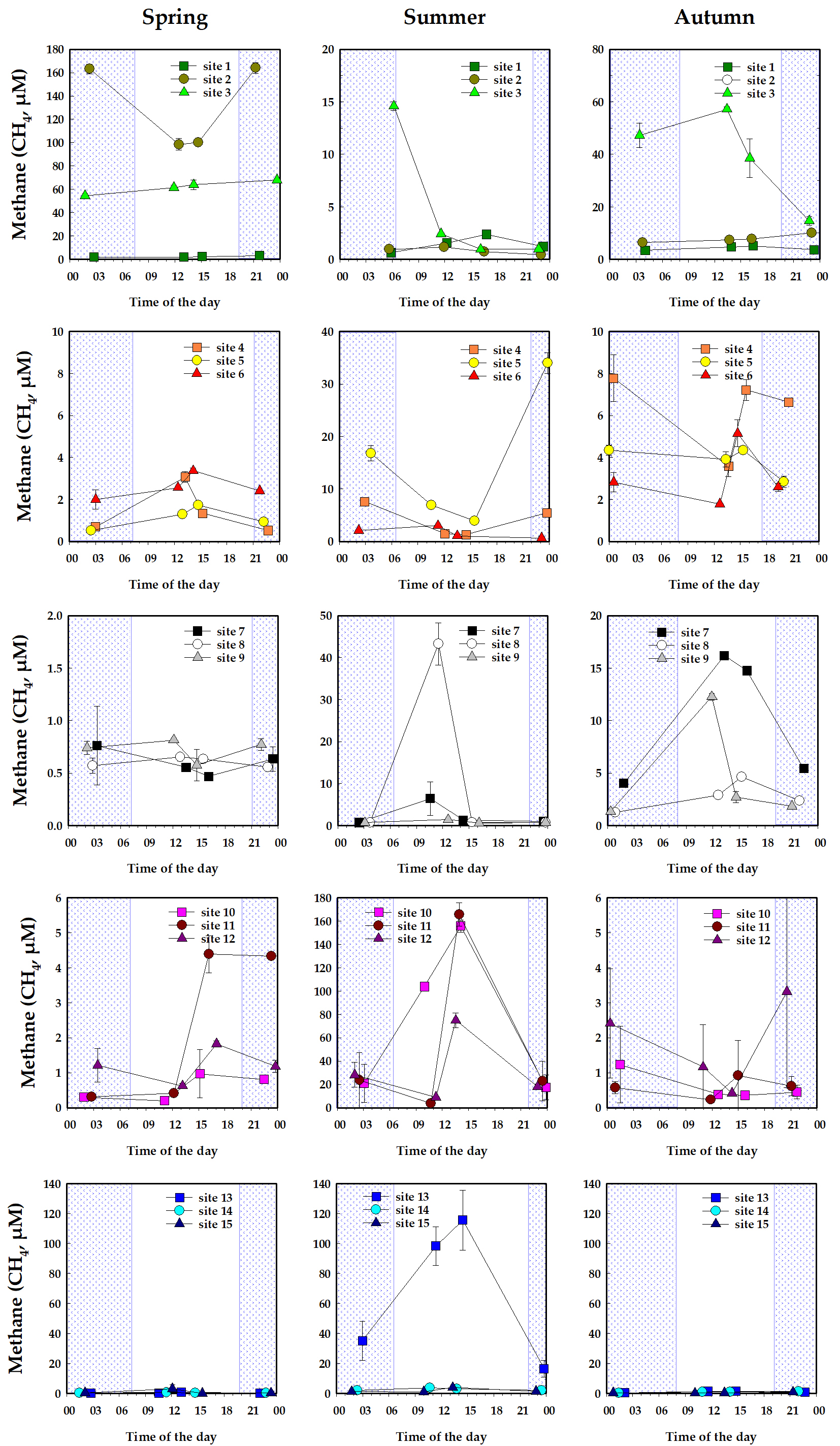

Supplement: Supplementary file 1 [file plants-10-01269-s001.zip › Figure S3.JPG]

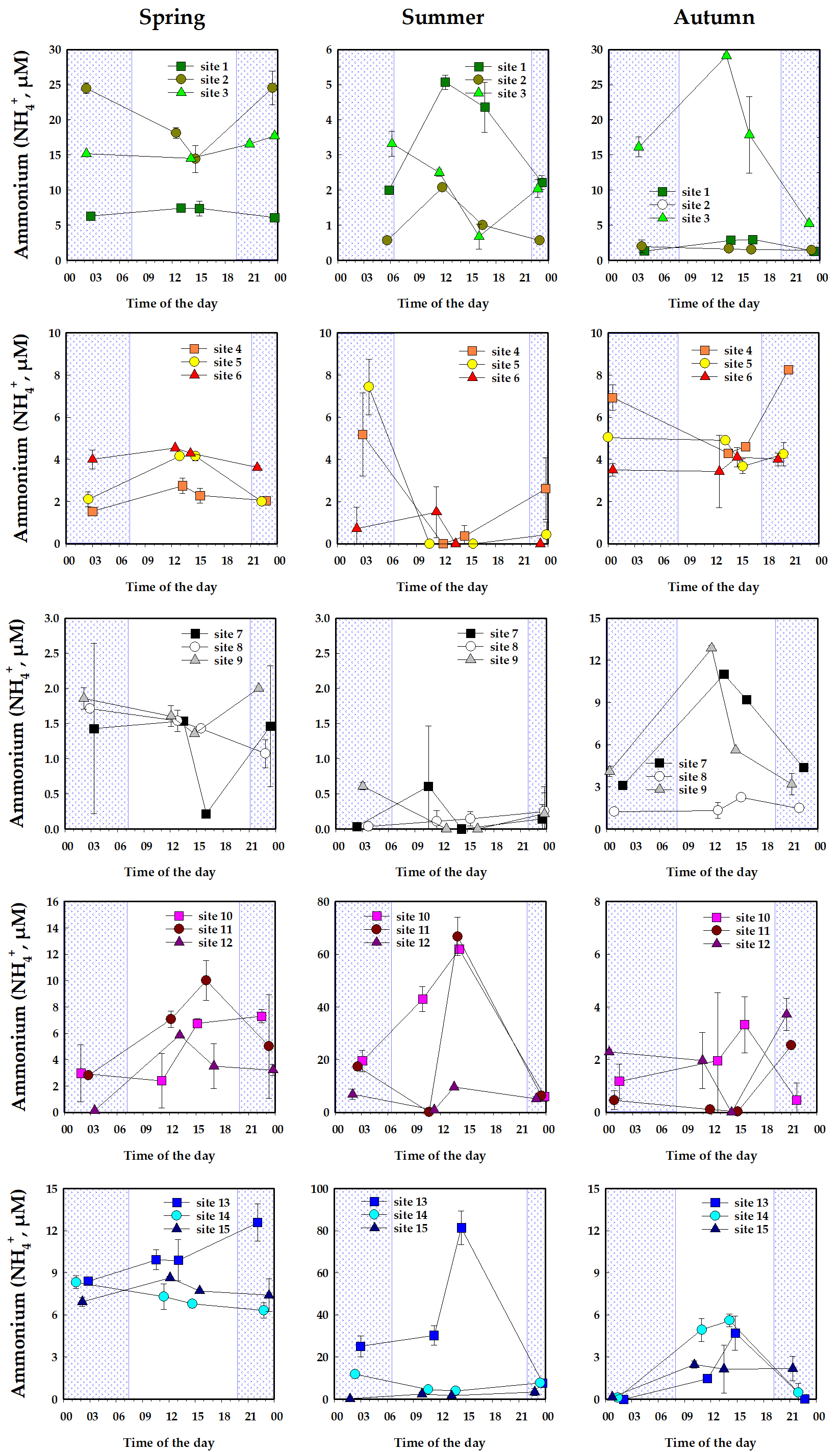

Supplement: Supplementary file 1 [file plants-10-01269-s001.zip › Figure S4.JPG]

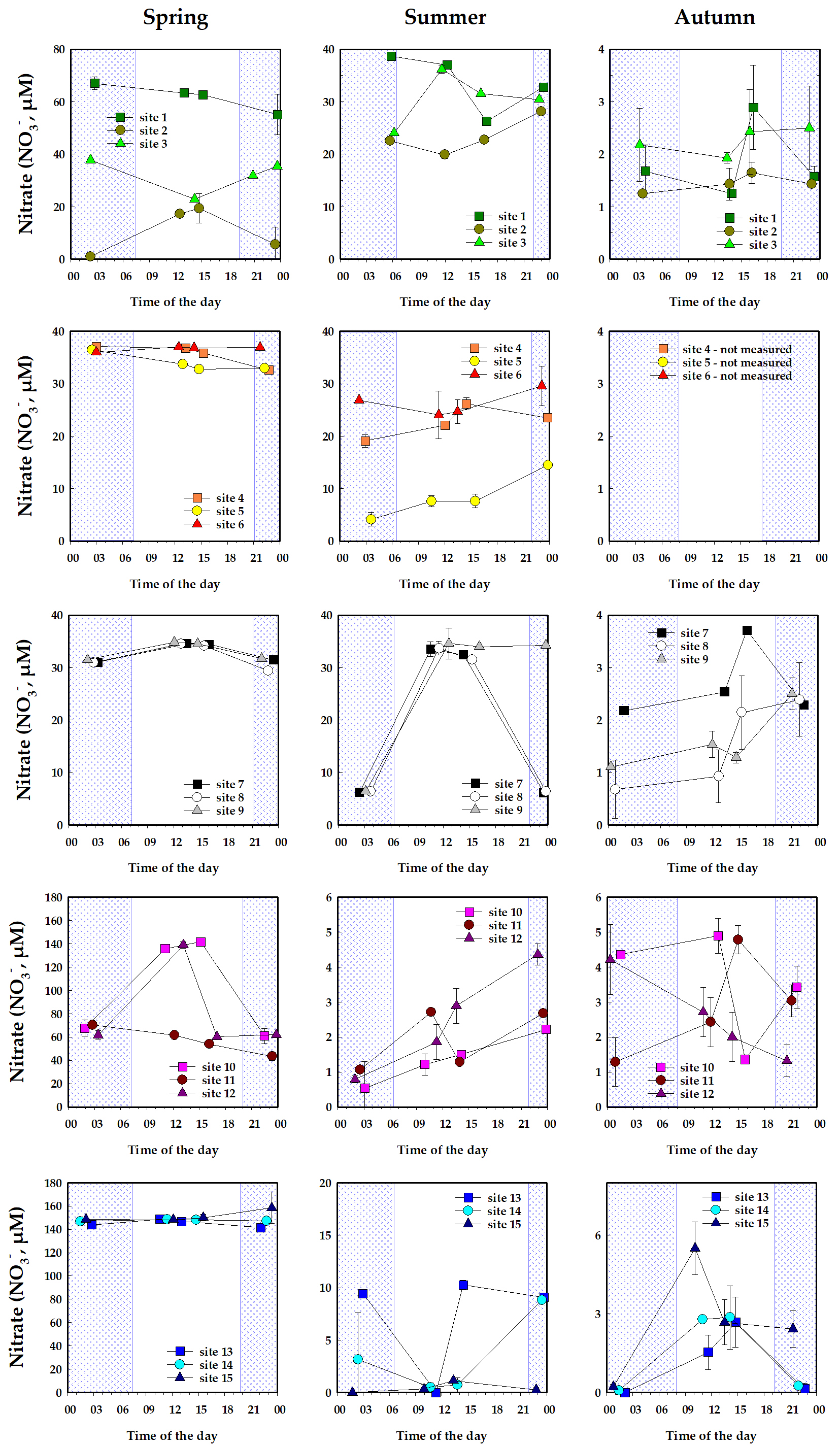

Supplement: Supplementary file 1 [file plants-10-01269-s001.zip › Figure S5.JPG]

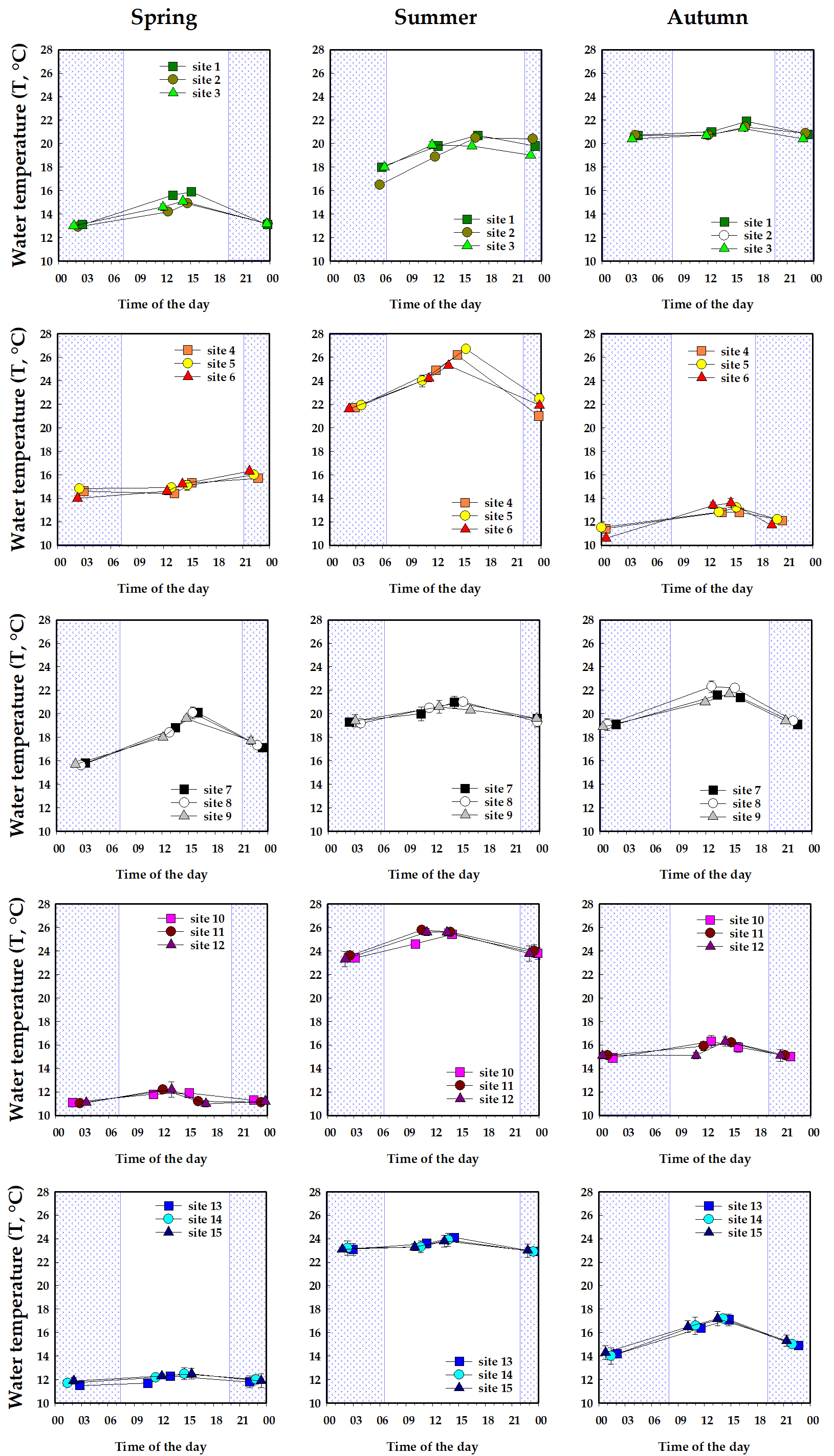

Supplement: Supplementary file 1 [file plants-10-01269-s001.zip › Figure S6.JPG]

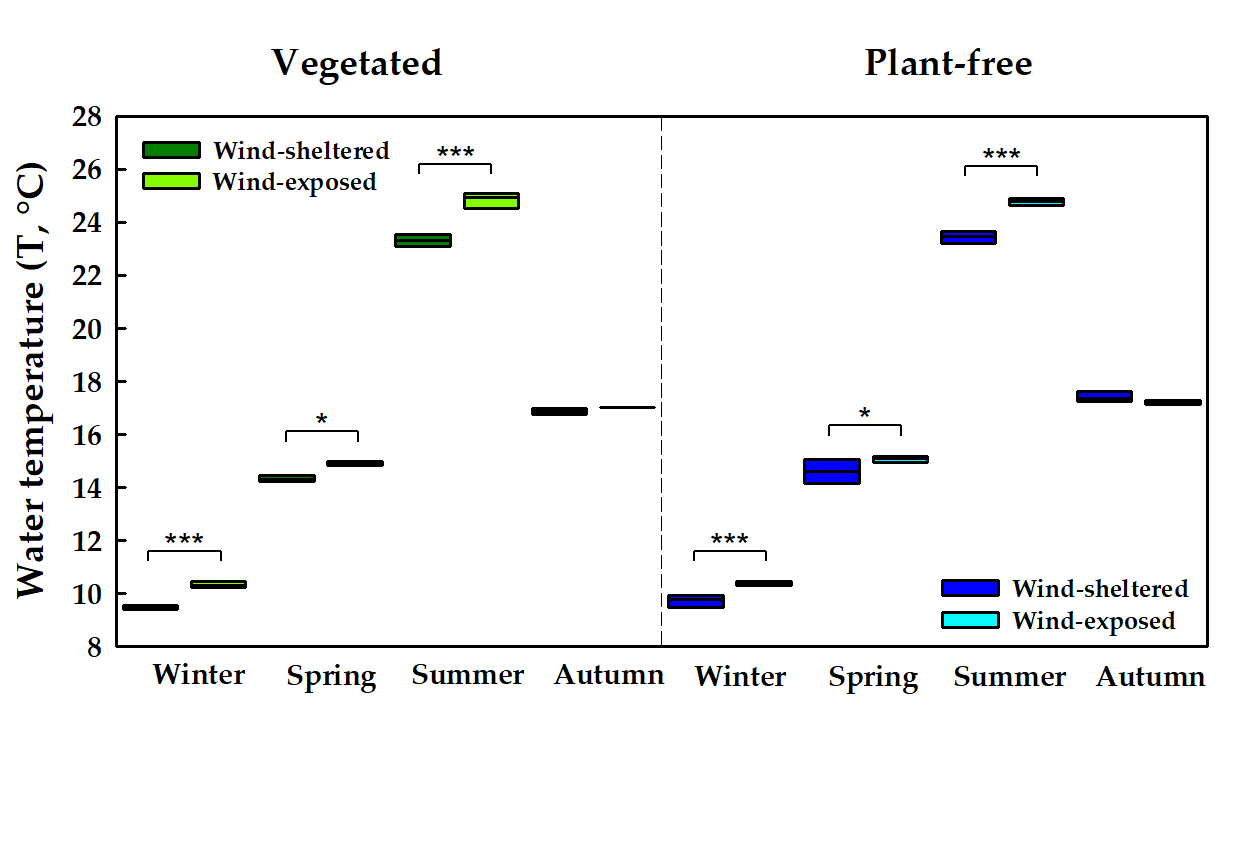

Supplement: Supplementary file 1 [file plants-10-01269-s001.zip › Figure S7.JPG]

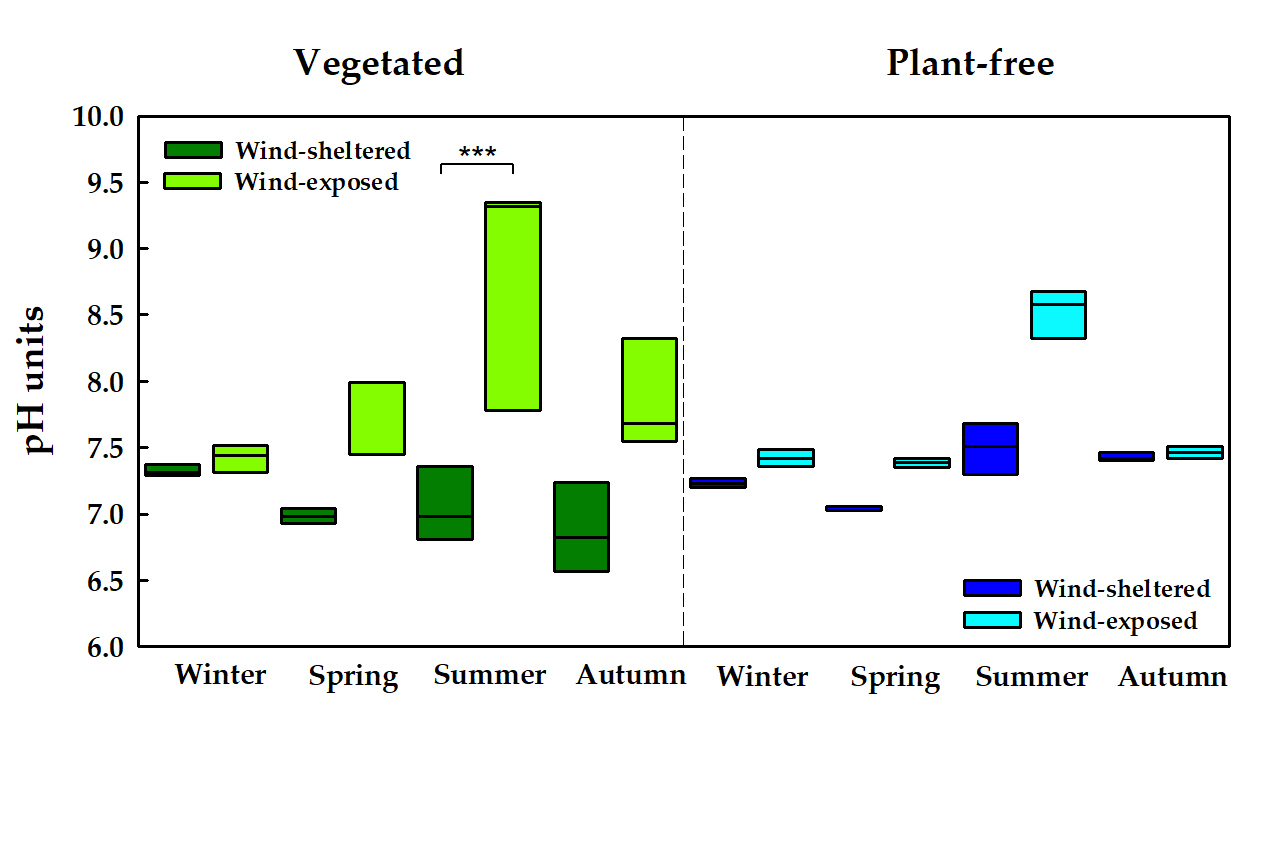

Supplement: Supplementary file 1 [file plants-10-01269-s001.zip › Figure S8.JPG]

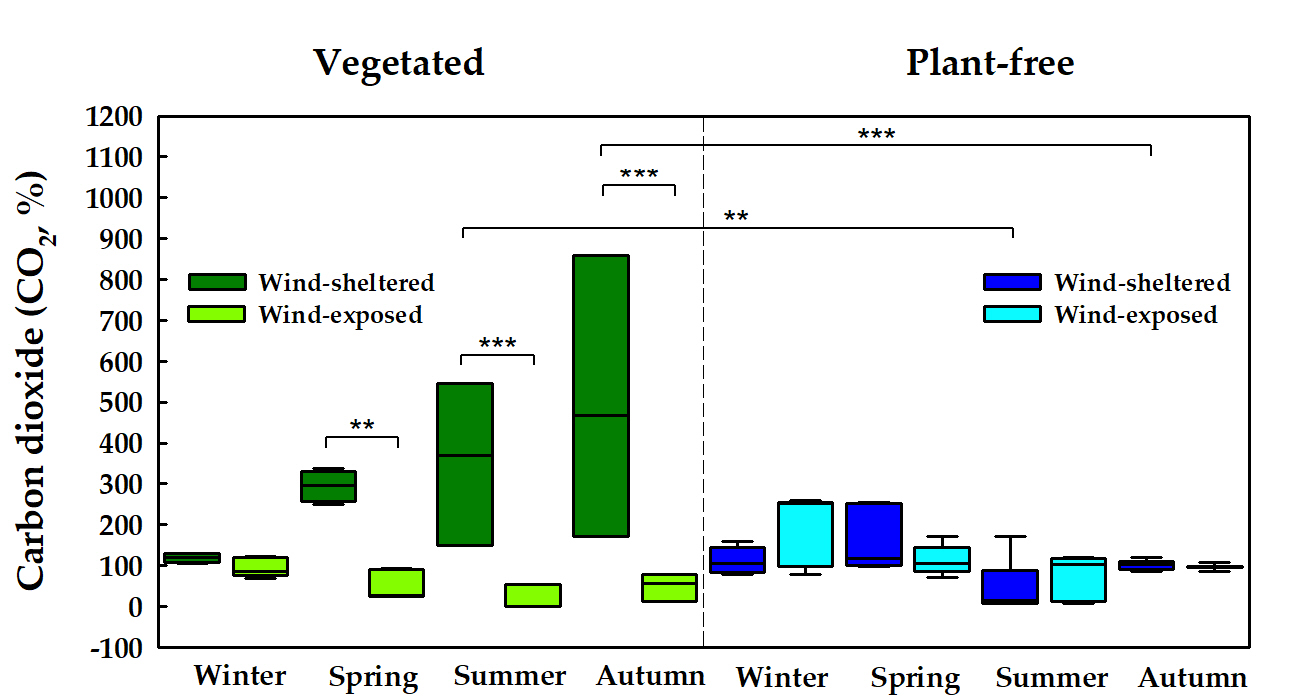

Supplement: Supplementary file 1 [file plants-10-01269-s001.zip › Figure S9.JPG]
